# The Breakdown in Training Mental Nurses

**Published:** 1921-02-26

**Authors:** 


### THE MATRONS' AND SISTERS' DEPARTMENT.

### THE BREAKDOWN IN TRAINING MENTAL NURSES.

It is a fact of great significance that the London County Council has been compelled to sanction a breach of the regulations, made in the interests alike of patients and staff, for the promotion of nurses to the posts of charge-nurse and charge night-nurse. According to the scheme laid down in conformity with the regulations of the Medico-Psychological Association, nurses are not promoted to these responsible posts until they have passed the prescribed examinations and have put in the prescribed term of service. Unfortunately, at the present time there is a great dearth in the mental hospitals under the London County Council, and also in the institutions of like nature under the Metropolitan Asylums Board, of probationers who have attained the required standard. The posts must be filled. Regulations have perforce given way, and some of the probationers promoted to be charge-nurses come considerably short of the minimum standard which the authorities in conclave determined upon.

We fear this is no mere temporary dislocation of the well-thought-out scheme devised by the Medico-Psychological Association for the training of mental nurses. It is a breakdown which, at the present moment, appears likely to affect very gravely the nursing of patients mentally afflicted. It affects the male mental nurse less seriously than the female. For the male asylum nurse embraces a career which he means to follow, at any rate for some years. The female probationer comes and goes. She is engaged by the week; she is paid what may be called, for a totally untrained girl of nineteen, a very high salary (under the M.A.B. it works out at £71 a year plus board, lodging and uniform); her hours on duty are limited to fifty a week. She has in some institutions the privilege of sleeping two nights out in the week, and has always one whole twenty-four hours off duty. And she responds to these advantages, less agreeably than might perhaps be expected, by regarding herself as a mere employee. She is frequently without interest in her duties, without desire to qualify herself for a higher post. She sets herself openly against study or passing any examination. In order to do this with more ease she is apt to leave the institution at the end of ten months when examinations are in the air, and sign on elsewhere, quite confident of being able to find a sufficient pretext for the change. Very often she takes up the work to fill in time before marriage and save a little for the furniture—a laudable proceeding, but one which, it must be admitted, is not likely to advance the cause of mental nursing.

It is very difficult to detect the causes or assign the remedy for this extremely unsatisfactory state of affairs. The Medico-Psychological Association has in the press a new set of regulations for the training and examination of mental nurses, and on these it may be possible to base a system which shall have the merit of attracting and retaining good candidates. The first step towards reform is to recognise that the present system of training nurses in mental hospitals and asylums is greatly at fault. It has proved unsuccessful, and new methods should be tried.

The position of the matron in mental hospitals is not always, in our judgment, what it ought to be. She appears often to lack that general responsibility for the training of the staff, their discipline and their supervision on and off duty, which rests on her compeers in general hospitals. The atmosphere and tone of a good training-school are sadly to seek in some mental hospitals. The influence is lacking which should permeate the entire establishment, and can only be set stirring through the matron and the trained sisters on whom she can rely, working through them in descending grades to the newest probationer. The proportion of the untrained is far too high. The result is routine work, without the ordered sequence of progress through which the probationer acquires interest in her profession and a keen desire to excel. Often the matron is the only trained nurse in the establishment, and is compelled, single-handed as it were, to keep the sick wards up to standard and train a large staff in the elements of sick-room duties. She needs competent assistants, well paid and well trained, some, though not necessarily all, from general hospitals. She needs the help of a sister-tutor able to inspire the reluctant probationers with interest in their studies. In short, the training-schools in many mental hospitals need thorough reorganisation.

